# The Relationships Between Trait Creativity and Resting-State EEG Microstates Were Modulated by Self-Esteem

**DOI:** 10.3389/fnhum.2020.576114

**Published:** 2020-11-11

**Authors:** Xin Wu, Jiajia Guo, Yufeng Wang, Feng Zou, Peifang Guo, Jieyu Lv, Meng Zhang

**Affiliations:** ^1^School of Psychology, Xinxiang Medical University, Xinxiang, China; ^2^Cognitive, Emotional, and Behavioral Lab, Xinxiang Medical University, Xinxiang, China; ^3^Department of Psychology, Central University of Finance and Economics, Beijing, China

**Keywords:** creativity, self-esteem, resting-state EEG, microstates, trait creativity

## Abstract

Numerous studies find that creativity is not only associated with low effort and flexible processes but also associated with high effort and persistent processes especially when defensive behavior is induced by negative emotions. The important role of self-esteem is to buffer negative emotions, and individuals with low self-esteem are prone to instigating various forms of defensive behaviors. Thus, we thought that the relationships between trait creativity and executive control brain networks might be modulated by self-esteem. The resting-state electroencephalogram (RS-EEG) microstates can be divided into four classical types (MS1, MS2, MS3, and MS4), which can reflect the brain networks as well as their dynamic characteristic. Thus, the Williams Creative Tendency Scale (WCTS) and Rosenberg Self-esteem Scale (RSES) were used to investigate the modulating role of self-esteem on the relationships between trait creativity and the RS-EEG microstates. As our results showed, self-esteem consistently modulated the relationships between creativity and the duration and contribution of MS2 related to visual or imagery processing, the occurrence of MS3 related to cingulo-opercular networks, and transitions between MS2 and MS4, which were related to frontoparietal control networks. Based on these results, we thought that trait creativity was related to the executive control of bottom-up processing for individuals with low self-esteem, while the bottom-up information from vision or visual imagery might be related to trait creativity for individuals with high self-esteem.

## Introduction

Creativity refers to the tendency to imagine and produce something novel (i.e., original) and unexpected, yet still appropriate (i.e., effective and useful) (Sternberg, 1999; Kaufman and Sternberg, 2010). In fact, creativity can be divided into those aspects related to personality and cognition (Rhodes, 1961; Gough, 1976; Amabile, 1996; Runco, 2007; Piffer, 2012). Williams (1969) suggested a cognitive–affective model of creativity and developed a corresponding creativity assessment packet (CAP) (Williams, 1969, 1980). The CAP included a divergent thinking (creative cognition) test and a divergent feeling (trait creativity) test (including four aptitude elements: imagination, risk taking, curiosity, and challenge) (Williams, 1993; Hwang et al., 2007; Liu et al., 2011). Based on previous findings, trait creativity is a set of aptitudes or personality variables that influence an individual’s creativity, while creative cognition refers to cognitive processes and metacognitive strategies during creative production, such as divergent thinking (Satzinger et al., 1999; Zeng et al., 2009). Although it is emphasized that creativity is a function of flexibility, creativity can be also achieved through persistence, which means that creative productions can be acquired by hard work, perseverance, and exploration of a few cognitive categories or perspectives (Schooler et al., 1993; Finke, 1996; Simonton, 1997; Dietrich, 2004; Nijstad et al., 2010).

Some cognitions of creativity had been confirmed by using divergent thinking tasks. It had been argued that generation of creative ideas require associative processes which include processes of freely and spontaneously forming associations between elements, as well as controlled processes which include inhibiting unsuitable ideas and evaluating and selecting creative ideas (Bendetowicz et al., 2017; Benedek et al., 2017). Beaty et al. (2017) also suggested that creative cognitions require the dynamic interactions between default and cognitive control networks, which reflected that both bottom-up and top-down processes are necessary to generate creative ideas. These opinions might fit with the blind variation and selective retention of creativity (Campbell., 1960; Simonton, 2011; Beaty et al., 2017), which implies the associative processes for blind variation and the controlled processes for selective retention. Other studies also found that the suppression of bottom-up irrelevant information is necessary when semantic information is retrieved and integrated to generate creative ideas (Fink et al., 2009, 2010; Wu et al., 2015). Sternberg (1999) suggested that trait creativity can have an impact on creative problem-solving ability. Individuals with certain creative traits (e.g., curiosity and imagination) can be more creative than those without these characteristics (Oldham and Cummings, 1996; Feist, 1998; Piffer, 2012). Moreover, creative individuals had higher gray matter volume in the right posterior middle temporal gyrus (related to representations of semantic concepts) (Li et al., 2015). On these perspectives, trait creativity might be related to the neural networks found in the creative cognitions to some extent.

Self-esteem is an attitude based on positive and negative self-evaluations (Rosenberg, 1965) and reflects the positive aspect of self-concept (Campbell et al., 1996). Individuals with high self-esteem tend to believe themselves to be capable and worthy, so they are more likely to express ideas that differ from others and are more willing to share creative ideas (Thatcher and Brown, 2010). The generative and flexible thinking associated with creativity can aid in successfully crafting self-serving justifications that allow individuals to maintain positive self-views (Carson et al., 2003; Gino and Ariely, 2012; Antinori et al., 2017). However, terror management theory suggests that the important role of self-esteem is to buffer anxieties induced by social threat, such as death threats (Greenberg et al., 1997; Pyszczynski, 2004) and negative feedback (Brown, 2010). Individuals with low self-esteem are prone to instigating various forms of defensive behavior to bolster their self-worth (Pyszczynski, 2004). Moreover, creativity can be achieved by persistence when defensive behavior is induced by negative mood states (such as fear and anxiety) (Baas et al., 2011; Roskes et al., 2012). Thus, creativity might be achieved by the function of flexibility for high-self-esteem individuals, while creativity might be achieved by the function of persistence for low-self-esteem individuals. It had been suggested that flexibility is associated with low effort, low resource demands, high speed, and efficient processing (Evans, 2003; Winkielman et al., 2003; Dietrich, 2004; De Dreu et al., 2008; Oppenheimer, 2008), while persistence is associated with high effort, perseverance, and a slower speed of operation (Evans, 2003; Winkielman et al., 2003; De Dreu et al., 2008). Thus, trait creativity might be related to controlled processes for individuals with low self-esteem, while it is related to associative processes for individuals with high self-esteem.

Previous studies had confirmed that functional networks can be depicted by spontaneous brain activities (Raichle and Mintun, 2006; Raichle, 2010), which might imply that the influence of self-esteem on trait creativity might be investigated by analyzing brain activity under the resting state. It had been found that some functional networks were confirmed by using the resting-state functional magnetic resonance imaging (RS-fMRI), such as default modal network, attentional network, salient network, and visual network (Andrews-Hanna et al., 2006; Raichle and Mintun, 2006; Fox et al., 2007). In addition, spontaneous brain activities are also investigated by the resting-state electroencephalogram (RS-EEG), where the RS-EEG microstates are used to depict the brain networks by using the signal from all electrodes (Dierks et al., 1997; Stevens and Kircher, 1998; Lehmann et al., 2005, 2010; Kikuchi et al., 2011; Schlegel et al., 2012). RS-EEG microstates are also seen as the “atoms of thought” and can be divided into four typical microstates (Lehmann et al., 1998; Khanna et al., 2014). When the evidences from RS-fMRI and RS-EEG are combined, the relevant brain networks are confirmed, which indicates that MS1 was related to the bilateral superior temporal gyrus and middle temporal gyrus, which were linked to semantic processes or phonological processing; MS2 was associated with the extrastriate cortex, which might be related to visual processing and visual images; MS3 was associated with positive BOLD activation in cingulo-opercular brain networks, which were related to salient or attention control; and MS4 was associated with right-lateralized dorsal and ventral attentional networks (Lehmann et al., 1987; Britz et al., 2010; Musso et al., 2010; Yuan et al., 2012).

Considering the high time resolution of EEG, RS-EEG microstates can also provide more dynamic characteristics of the brain networks relative to RS-fMRI. Specifically, duration is the time coverage of each microstate; occurrence is the average number of occurrences per microstate in a second; contribution is the total duration of each microstate, accounting for the total resting EEG duration; the possibility of transition between any two microstates is related to the information flow between them (Britz et al., 2010; Khanna et al., 2014; Gao et al., 2017). Moreover, the characteristics are related to the altered mental states under experimental conditions. Seitzman et al. (2017) found that the occurrence and contribution of MS2 and the duration of MS1 were modulated by the eye-open or eye-close condition; the occurrence and contribution of MS4 were increased under attentional tasks; the transition between MS3 and MS1 was also decreased under attentional tasks. Zappasodi et al. (2019) also found that the microstates related to visual (MS2) and default-mode network (MS3) were modulated by visuospatial tasks, which reflect that the contribution of MS2 was significantly increased under visuospatial tasks, while the contribution of MS2 was significantly decreased. Moreover, Santarnecchi et al. (2017) found that the RS-EEG microstates of MS2 and MS3 were related to fluid intelligence, where they found that the occurrences of MS2 and MS3 were significantly negatively related to fluid intelligence, and the contribution of MS2 was negatively associated with the increase of fluid intelligence after training it.

According to previous studies (De Dreu et al., 2008; Bendetowicz et al., 2017; Benedek et al., 2017), we speculated that trait creativity for individuals with low or high self-esteem might rely on different cognitive processes. Specifically, trait creativity might be related to the controlled processes for individuals with low self-esteem, while it is related to associative processes for individuals with high self-esteem. Thus, relationships between trait creativity and RS-EEG microstates might be modulated by self-esteem. In the present study, the modulated roles of self-esteem in the relationships between creativity and RS-EEG microstates were investigated by using the Williams Creative Tendency Scale (WCTS) and Rosenberg Self-esteem Scale (RSES) to measure the creativity and self-esteem, respectively, and combining the RE-EEG microstate analysis. Previous studies considered that the RS-EEG microstates were calculated based on the alpha band activities and with the inhibition of modality-specific processing, increasing the characteristics of MS2 and MS3 (Milz et al., 2016; Santarnecchi et al., 2017). Thus, we hypothesize that the temporal characteristics of sensory input (such as MS1 and MS2) might be positively related to trait creativity for individuals with low self-esteem, which make them inhibit bottom-up irrelevant information. However, these characteristics might be negatively related to trait creativity for individuals with high self-esteem, which make them generate more associations. In addition, the possibility of transitions between top-down control system (MS3 or MS4) and sensory input (such as MS1 and MS2) might be higher for those with low self-esteem relative to high self-esteem, which makes it easier for them to control the bottom-up irrelevant information.

## Materials and Methods

### Subjects

Three hundred thirty-six right-handed subjects recruited in Xinxiang Medical University (72% male, 28% female; mean age = 18.3, SD = 0.84) participated in the study. Subjects had no history of neurological or psychiatric disease and did not take any medication that could affect the experiment. All participants gave written informed consent to participate in the study which was approved by the ethics committee of Xinxiang Medical University. One subject’s data were deleted due to data record error.

### Materials

#### Williams Creative Tendency Scale

The WCTS was used to measure trait creativity (revised by Lin Xingtai of Taiwan Normal University). The WCTS is composed of 50 items, and the subjects were asked to respond to a 3-point Likert-type scale ranging from 1 (totally disagree) to 3 (totally agree). According to Williams (1994), WCTS can be divided into four subscales, namely, curiosity (13 items; e.g., “I would like to know what other people think”), imagination (13 items; e.g., “If the final page of a storybook is missing, I will make up the story’s ending myself”), challenge (12 items; e.g., “I like unusual things”), and risk taking (12 items; e.g., “Trying a new game or activity is an interesting thing”). Reliability analysis showed that the reliability coefficients of the total score of the scale were between 0.569 and 0.678. In this study, the alpha reliability for the WCTS was 0.866 according to our sample.

#### Rosenberg Self-Esteem Scale

Participants completed a measure of self-esteem: RSES (Rosenberg, 1965). The scale is a self-assessment measure of self-esteem commonly used at home and abroad, which consists of 10 items. All 10 items are rated on a 4-point scale ranging from 1 (not very true of me) to 4 (very true of me). On a scale of 10 to 40, higher scores indicate higher levels of self-esteem and self-acceptance. Previous studies have reported alpha reliability for the RSES ranging from 0.72 to 0.88 (Gray-Little and Carels, 1997). In this study, the alpha reliability for the RSES was 0.816 according to our sample.

### RS-EEG Data Acquisition

During RS-EEG recording (6 min in duration), subjects were asked to open their eyes and focus on the “+” appearing in the center of the screen quietly without moving their body or head. The RS-EEG data were recorded by using the Neuro Scan Product with 64 Ag-AgCl scalp sites according to the international 10–20 system in an elastic cap. During recording, all electrodes were referenced to Cz and re-referenced off-line to linked mastoids. Channels for horizontal and vertical EEG were computed off-line from electrodes recorded from the outer canthi of the eyes and from above and below the right eye, respectively. Electrode impedance was kept below 5 kΩ. EEG was sampled online with a frequency of 500 Hz DC amplifiers with a band-pass filter of 0.1–100 Hz.

### RS-EEG Microstate Preprocessing

The EEG data were preprocessed using EEGLAB^1^ in MATLAB 2018b^2^. Data were filtered off-line by a band-pass filter of 2–20 Hz and were run through an independent component analysis (ICA). Artifacts produced by blinks, eye movements, eye drift, head movements, power-line interference, or electrocardiograph were rejected. The artifact-free data were recomputed against the average, according to previous studies (Lehmann et al., 1987, 2005; Koenig et al., 1999; Gao et al., 2017). Then the data were segmented into 180 epochs with an epoch length of 2,000 ms. EEG epochs with amplitude values exceeding ± 80 μV, which might be contaminated by strong muscle artifacts, were manually rejected.

**FIGURE 1:**
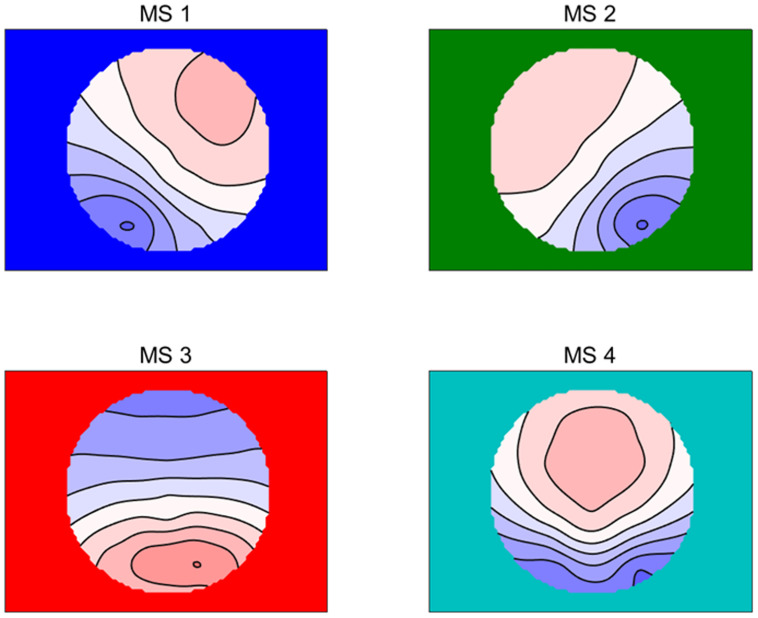
The four microstate topographic maps are RS-EEG microstate Type A (MS1), Type B (MS2), Type C (MS3), and Type D (MS4).

The RS-EEG microstates were calculated according to previous studies (Lehmann et al., 1987, 2005; Koenig et al., 1999; Gao et al., 2017). First, the global field power (GFP), which was defined as the EEG potential variance across scalp electrodes, was calculated, and only the topographies at peaks of the GFP were further analyzed. Then, based on previous studies (Tibshirani and Walther, 2005), atomize–agglomerate hierarchical clustering (AAHC) was performed to analyze the microstates with the polarity of each topographical map being disregarded, which was a modified *k*-means to create unique clusters for EGG microstate analysis. After that, a cross-validation criterion was used to identify the optimal number of template maps, which was the best solution to find the minimal number of template maps explaining the maximal variance in cluster analysis (Pascual-Marqui et al., 1995; Britz et al., 2010). According to our data, four clusters (MS1, MS2, MS3, and MS4) were found, and the explained variance was 0.786 ± 0.033 (see Figure 1), which was the same as that found in most studies of RS-EEG microstate (e.g., Lehmann et al., 1987, 2005; Britz et al., 2010; Gao et al., 2017). The global map dissimilarity (GMD) was used as a criterion to fit all original maps of each subject into the four prototype maps, where each time point was fitted and labeled with the one cluster it correlated best (Van de Ville et al., 2010). Finally, the labeled data were used to compute the temporal characteristics, namely, duration, occurrence, and contribution of each microstate, as well as the probability of transition between them.

### Statistical Analysis

The total scores of the WCTS and RSES were imported into SPSS for correlation analysis, and the correlation between creativity and self-esteem as well as the correlation between the four dimensions of creativity (risk taking, challenge, curiosity, and imagination) and self-esteem was obtained. Then, the total score of the scales (WCTS and RSES) and the data of the duration, occurrence, and contribution of four RS-EEG microstates were imputed into SPSS to analyze the relationship between creativity and microstates and between self-esteem and microstates. Finally, the modulating role of RSES in the relationship between the total score of WCTS and the duration, occurrence, and contribution of microstates and the transitions between them were analyzed using Model 1 of PROCESS 3.0 (Hayes, 2018) with a statistical threshold of *p* < 0.05 (FDR corrected).

## Results

### The Relationship Between Self-Esteem and Creativity

**FIGURE 2:**
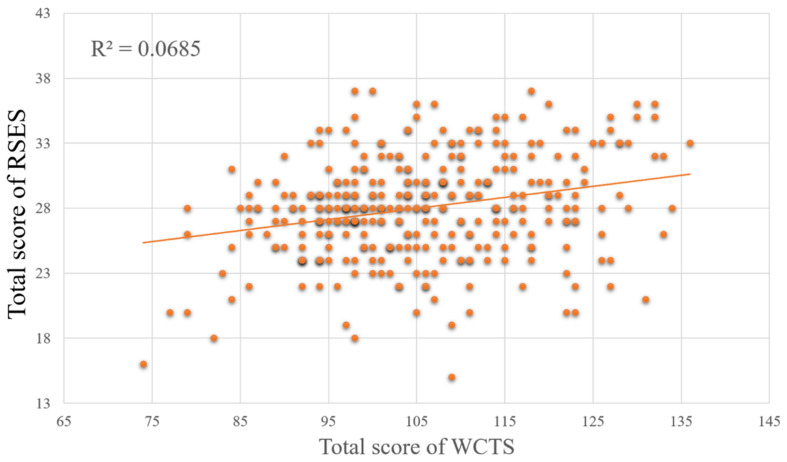
The relationships between the total score of WCTS and total score of RSES.

Since some data were more than three standard deviations from the mean, we chose to exclude them and ended up with data from 334 subjects available. The total score of RSES is significantly positively correlated with the total score of WCTS (*r* = 0.262, *p* < 0.001) (see Figure 2), as well as the four subscales of WCTS [risk taking (*r* = 0.294, *p* < 0.001), challenge (*r* = 0.316, *p* = 0.001), and curiosity (*r* = 0.186, *p* < 0.001), imagination (*r* = 0.112, *p* < 0.05)] (see Table 1). In addition, the original score of the AU task was not significantly correlated to the total score of RSES.

**TABLE 1:** The relationships between self-esteem and creativity (*n* = 334).

	**Risk taking**	**Curiosity**	**Imagination**	**Challenge**	**Creativity**
SES	0.2949 (<0.001)	0.186 (0.001)	0.112 (0.040)	0.316 (<0.001)	0.262 (<0.001)

*The bolded and italic values mean that the relationship was significant at *p* < 0.05 with Bonferroni correction.*

### The Relationship Between Self-Esteem, Creativity, and Microstates

Through the correlation analysis of creativity and its different dimensions with various types of microstates, we found no significant correlation between creativity and microstates. On a regular basis, we also analyzed the relationship between self-esteem and various types of microstates; however, there was no significant correlation. Further analysis had shown that age and gender had no effect on the relationships.

### The Modulating Role of RSES in Creativity of WCTS

**FIGURE 3:**
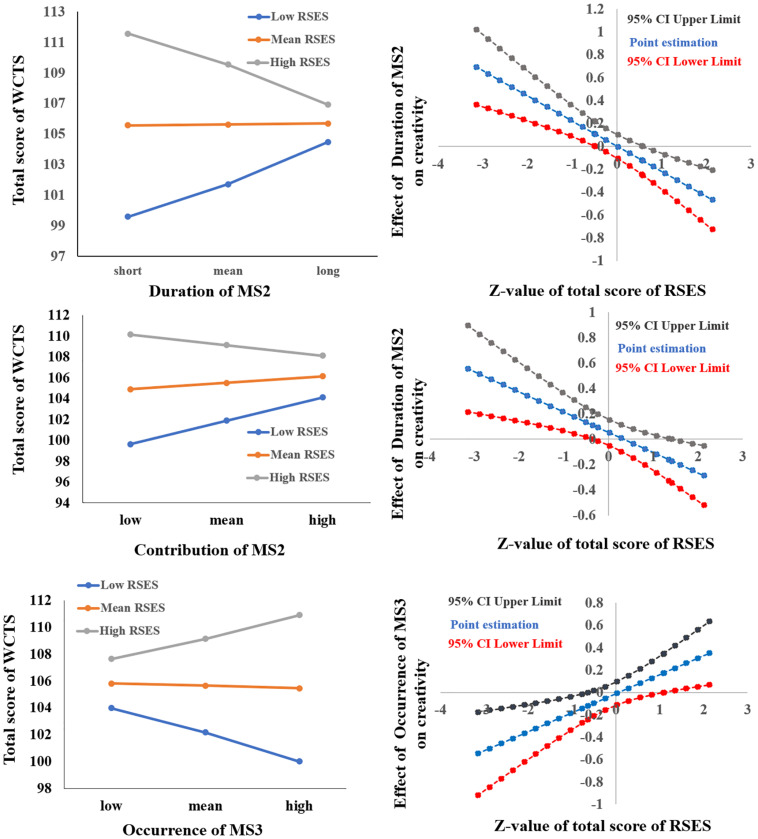
The relationships between parameters RS-EEG microstates (duration and contribution of MS2 and occurrence of MS3) and total score of WCTS were modulated by total score of RSES.

After sex and age were controlled for, the interaction of the total score of RSES × the mean duration of MS2 was significant [*F*(1,329) = 17.691, *p* < 0.001, Δ*R*^2^ = 0.046] (see Table 2). Simple slope analysis results showed that when the total RSES was lower (mean – 1 sd), the total score of WCTS was significantly positively correlated with the duration of MS2 (β = 0.218, *t* = 3.192, *p* < 0.005) and that when the total RSES score was higher (mean + 1 sd), the total score of WCTS was significantly negatively correlated with the duration of MS2 (β = −0.204, *t* = −2.650, *p* < 0.01). Johnson–Neyman results showed that the total score of WCTS was significantly positively correlated with the duration of MS2 when the total score of RSES was below 27 (mean –0.486 sd, 31.04% of our sample) and that the total score of WCTS was significantly negatively correlated with the duration of MS2 when the total score of RSES was above 30 (mean + 0.580 sd, 23.88% of our sample) (see Figure 3).

**TABLE 2:** The interaction parameters of RS-EEG microstate and total score of RSES when predicting total score of WCTS after controlling sex and age.

	**△*R*^2^**	**df1**	**df2**	***F*-value**	***p* (Uncorrected)**	***p* (FDR-corrected)**
**Duration**						
MS1	0.004	1	329	1.548	0.214	0.278
**MS2**	**0.046**	**1**	**329**	**17.691**	**< 0.001**	**0.005**
MS3	0.007	1	329	2.440	0.119	0.226
MS4	0.007	1	329	2.411	0.122	0.226
**Occurrence**						
MS1	0.014	1	329	5.090	0.024	0.082
MS2	0.002	1	329	0.060	0.807	0.807
**MS3**	**0.022**	**1**	**329**	**8.061**	**0.005**	**0.030**
MS4	0.012	1	329	4.372	0.037	0.111
**Contribution**						
MS1	0.007	1	329	2.397	0.123	0.226
**MS2**	**0.026**	**1**	**329**	**9.598**	**0.002**	**0.024**
MS3	0.003	1	329	1.122	0.290	0.316
MS4	0.001	1	329	0.299	0.585	0.610
**Transition**						
MS1 to MS2	0.005	1	329	1.623	0.204	0.278
MS1 to MS3	0.004	1	329	1.274	0.260	0.297
MS1 to MS4	0.016	1	329	5.887	0.016	0.064
MS2 to MS1	0.004	1	329	1.514	0.220	0.278
MS2 to MS3	0.004	1	329	1.295	0.256	0.297
**MS2 to MS4**	**0.023**	**1**	**329**	**8.652**	**0.004**	**0.030**
MS3 to MS1	0.009	1	329	3.178	0.076	0.182
MS3 to MS2	0.006	1	329	2.202	0.139	0.226
MS3 to MS4	0.005	1	329	1.633	0.202	0.278
MS4 to MS1	0.010	1	329	3.739	0.054	0.144
**MS4 to MS2**	**0.018**	**1**	**329**	**6.766**	**0.009**	**0.047**
MS4 to MS3	0.006	1	329	2.179	0.141	0.226

*The bold values are significant in *p* < 0.05 with FDR-corrected.*

After sex and age were controlled for, the interaction of the total score of RSES × the mean contribution of MS2 was significant [*F*(1,329) = 9.598, *p* < 0.005, Δ*R*^2^ = 0.026] (see Table 2). Simple slope analysis results showed that when the total RSES was lower (mean – 1 sd), the total score of WCTS was significantly positively correlated with the contribution of MS2 (β = 0.211, *t* = 2.835, *p* = 0.005) and that when the total RSES score was higher (mean + 1 sd), the total score of WCTS was not significantly correlated with the contribution of MS2 (β = −0.095, *t* = −1.376, *p* = 0.170). Johnson–Neyman results showed that the total score of WCTS was significantly positively correlated with the contribution of MS2 when the total score of RSES was below 27 (mean – 0.486 sd, 31.04% of our sample) and that the total score of WCTS was significantly negatively correlated with the contribution of MS2 when the total score of RSES was above 32 (mean + 1.414 sd, 14.63% of our sample) (see Figure 3).

After sex and age were controlled for, the interaction of the total score of RSES × the mean occurrence of MS3 was significant [*F*(1,329) = 8.061, *p* = 0.005, Δ*R*^2^ = 0.022] (see Table 2). Simple slope analysis results showed that when the total RSES was lower (mean – 1 sd), the total score of WCTS was significantly negatively correlated with the occurrence of MS3 (β = −0.179, *t* = −2.417, *p* < 0.05); when total RSES was higher (mean + 1 sd), the total score of WCTS was not significantly correlated with the occurrence of MS3 (β = 148, *t* = −2.417, *p* = 0.070). Johnson–Neyman results showed that the total score of WCTS was significantly negatively correlated with the occurrence of MS3 when the total score of RSES was below 25 (mean – 0.643 sd, 17.61% of our sample) and that the total score of WCTS was significantly positively correlated with the occurrence of MS3 when the total score of RSES was above 32 (mean + 1.414 sd, 14.63% of our sample) (see Figure 3).

**FIGURE 4:**
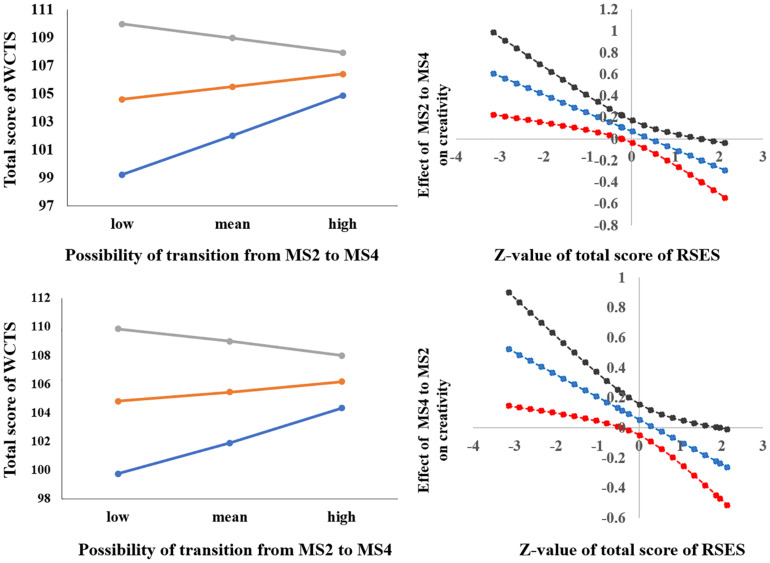
The relationships between parameters RS-EEG microstates (transition from MS2 to MS4 and transition from MS4 to MS2) and total score of WCTS were modulated by total score of RSES.

After sex and age were controlled for, the interaction of the total score of RSES × the possibility of transition from MS2 to MS4 [possibility _(MS__2__to MS__4__)_] was significant [*F*(1,330) = 10.122, *p* < 0.005, Δ*R*^2^ = 0.028] (see Table 2). Simple slope analysis results showed that when the total RSES was lower (mean – 1 sd), the total score of WCTS was significantly positively correlated with possibility _(MS__2__to MS__4__)_ (β = 0.239, *t* = 2.952, *p* < 0.005) and that when total RSES was higher (mean + 1 sd), the total score of WCTS was not significantly correlated with possibility _(MS__2__to MS__4__)_ (β = −0.087, *t* = −1.217, *p* = 0.224). Johnson–Neyman results showed that the total score of WCTS was significantly positively correlated with the duration of MS2 when the total score of RSES was below 27 (mean – 0.486 sd, 31.04% of our sample) and that the total score of WCTS was significantly negatively correlated with the duration of MS2 when the total score of RSES was above 32 (mean + 1.414 sd, 14.63% of our sample) (see Figure 4).

After sex and age were controlled for, the interaction of the total score of RSES × the possibility of transition from MS4 to MS2 [possibility _(MS__4__to MS__2__)_] was significant [*F*(1,329) = 6.766, *p* < 0.01, Δ*R*^2^ = 0.018] (see Table 2). Simple slope analysis results showed that when the total RSES was lower (mean – 1 sd), the total score of WCTS was significantly positively correlated with possibility _(MS__4__to MS__2__)_ (β = −0.203, *t* = 2.527, *p* < 0.05) and that when total RSES was higher (mean + 1 sd), the total score of WCTS was not significantly correlated with possibility _(MS__4__to MS__2__)_ (β = −0.082, *t* = −1.151, *p* = 0.251). Johnson–Neyman results showed that the total score of WCTS was significantly negatively correlated with the duration of MS2 when the total score of RSES was below 27 (mean – 0.405 sd, 31.04% of our sample) and that the total score of WCTS was significantly negatively correlated with the duration of MS2 when the total score of RSES was above 36 (mean + 1.956 sd, 2.39% of our sample) (see Figure 4).

## Discussion

In the present study, the modulating role of self-esteem in the relationship between creativity and RS-EEG microstates was investigated using WCTS and RSES combined with RS-EEG microstate analysis. Consistent with the previous studies, this experiment also proved the positive correlation between trait creativity and self-esteem (Jaquish and Ripple, 1981; Goldsmith and Matherly, 1988; Yau, 2011). Importantly, the RS-EEG microstate results showed that RSES could modulate the relationship between WCTS creativity and the duration and contribution of MS2, the occurrence of MS3, and the possibility _(MS__4__to MS__2__)_.

In the previous series of studies, it had been suggested that a wide breadth of attention could facilitate creative performance (Mendelsohn and Griswold, 1964, 1966; Mendelsohn and Lindholm, 1972; Mendelsohn, 1976), which means that the greater the number and range of stimuli attended to at any one time, the more chances there are to generate creative ideas (Kasof, 1997; Memmert and Roth, 2007). Previous studies from visual attention found that the activation of the visual cortex (striated and extrastriated cortex) could be influenced by visual attention, which means that the activation of the visual cortex can be modulated by operating both through the facilitation of visual processing at the attended location and through inhibition of unattended stimulus representations (Slotnick et al., 2003). Moreover, it had been found that the extrastriate cortex was also activated when creative tasks (such as alternative uses) were performed (Fink et al., 2009, 2010) and that gray matter density in the visual cortex was positively correlated with creativity (Fink et al., 2014; Wu et al., 2015). Previous studies had found that MS2 was negatively associated with the activation of the extrastriate cortex, which might imply that individuals with a short duration of MS2 possess a stronger function of visual processing or visual images (Britz et al., 2010; Khanna et al., 2015; Gao et al., 2017). Therefore, for individuals with higher self-esteem, the duration of MS2 was negatively correlated with the total score of WCTS, which might reflect that a strong function of the visual cortex could make individuals attend more elements at one time and make individuals more creative.

According to the model of a dual pathway to creativity, creative ideas can be generated by the functions of flexibility and persistence (Nijstad et al., 2010). It had been suggested that inhibition of irrelevant bottom-up cognitive processes was required for creativity (e.g., Fink et al., 2009, 2010; Wu et al., 2015), especially when the function of persistence was induced under threat conditions (Baas et al., 2011; Roskes et al., 2012). According to terror management theory (Greenberg et al., 1997) and sociometer theory (Leary et al., 1995), the important role of self-esteem is to buffer negative emotions induced by death threats (death anxiety) or social threats (social rejection). Thus, bottom-up cognitive processes might be a disadvantage to creativity for individuals with low elf-esteem. Consistent with this opinion, our results showed that the duration of MS2 was positively correlated with the total score of WCTS when individuals have low self-esteem. Therefore, we thought that irrelevant bottom-up cognitive processes might be more prone to being inhibited as the duration of MS2 increases, which is good for individuals with low self-esteem as this enables them to generate creative ideas.

Now that inhibition of irrelevant bottom-up cognitive processes is required for creativity (e.g., Fink et al., 2009, 2010; Wu et al., 2015), the transitions between MS4 (executive control) and MS2 (visual processes) found in this study might also reflect that persistence is needed for creativity in individuals with low self-esteem. Previous studies had found that MS4 was related to right-lateralized frontoparietal networks, which might be related to dorsal and ventral attention networks (Britz et al., 2010). It had been confirmed that the right dorsal frontal–parietal networks were involved in top-down control, while the ventral frontal–parietal networks were related to information-capture attention in the bottom-up manner (Cabeza et al., 2008, Cabeza et al., 2014). It was further found that the ventral frontal–parietal networks were related to the phasic and adaptive aspects of cognitive control (moment-to-moment executive control), while dorsal frontal–parietal networks were related to top-down selective attention to specific stimulus features (Sadaghiani et al., 2010, 2012; Sadaghiani and Kleinschmidt, 2016). According to our results, the possibility of transitions between MS2 and MS4 was positively correlated to the total score of WCTS for individuals with low self-esteem; at the same time, the possibility of transition from MS2 to MS4 was negatively correlated to the total score of WCTS for individuals with high self-esteem. Thus, the trait creativity for individuals with low self-esteem might be dependent on the moment-to-moment information to attention in a bottom-up manner, but the trait creativity for individuals with high self-esteem might be dependent on the top-down selective attention to specific stimulus features.

The pursuit of self-esteem is a fundamental human need (Taylor and Brown, 1988; Solomon et al., 1991), but the consequences of pursuing self-esteem may produce the risk of failure in verifying individuals’ abilities, qualities, and self-worth and make them experience uncertainty (Crocker and Park, 2004). Moreover, individuals who chronically experience real or imagined rejection are prone to developing lower self-esteem relative to individuals feeling accepted and included in their social environment (Dandeneau and Baldwin, 2004). It had been found that the dorsal anterior cingulate cortex (dACC), the right ventral lateral prefrontal cortex (rVLPFC), and the anterior insular (AI) were more active during rejection than during inclusion (Eisenberger et al., 2003, 2007; Slavich and Epel, 2010; Masten et al., 2011; Rotge et al., 2014). Moreover, RS-EEG MS3 was related to the cingulo-opercular networks, which include the dACC and insula (Katayama et al., 2007; Seeley et al., 2007; Britz et al., 2010). Therefore, the negative effect of occurrence of RS-EEG MS3 on the trait creativity for individuals with low self-esteem might reflect that with the functions of cingulo-opercular networks increasing, individuals might be prone to being influenced by social threat and make them develop lower trait creativity.

This experiment investigated the modulating effect of self-esteem on creativity and RS-EEG microstates. The findings suggest that self-esteem modulates the relationship between creativity and the duration and contribution of MS2, the occurrence of MS3, and the possibility _(MS__4__to MS__2__)_. Based on these results, we thought trait creativity was related to automatic or bottom-up cognitive processes for individuals with high self-esteem, while inhibition of irrelevant information could facilitate creativity for individuals with low self-esteem. Moreover, social threat experiences might have a detrimental effect on creativity for individuals with low self-esteem. Though there were some important and robust evidences for us to understand the relationships between creativity and RS-EEG microstates, several limitations should be considered. Firstly, only sex and age were controlled, and some other potential factors for creativity (such as intelligence and personality) were not controlled. Secondly, complex cognitive processes could be related to creativity; however, only some of them have been reflected by our results. Thirdly, due to undergraduates being selected in this study, it might be cautious to explore other groups with different ages (such as children and old adults). Therefore, more detailed experiments and advanced paradigms should be used in future studies to determine the cognitive meanings of each microstate to further investigate the relationships between microstates and creativity.

## Data Availability Statement

The raw data supporting the conclusions of this article will be made available by the authors, without undue reservation.

## Ethics Statement

The studies involving human participants were reviewed and approved by Xinxiang Medical University ethics committee. The patients/participants provided their written informed consent to participate in this study.

## Author Contributions

XW, JG, and MZ designed the experiment. JG wrote this manuscript. YW, FZ, and PG collected and analyzed the data. XW and MZ revised a manuscript. All authors contributed to the article and approved the submitted version.

## Conflict of Interest

The authors declare that the research was conducted in the absence of any commercial or financial relationships that could be construed as a potential conflict of interest.

## Footnotes

1 https://sccn.ucsd.edu/eeglab/index.php

2 http://cn.mathworks.com/
